# The Kok effect revisited

**DOI:** 10.1111/nph.16638

**Published:** 2020-06-03

**Authors:** Xinyou Yin, Yuxi Niu, Peter E. L. van der Putten, Paul C. Struik

**Affiliations:** ^1^ Centre for Crop Systems Analysis Department of Plant Sciences Wageningen University & Research PO Box 430 Wageningen 6700 AK the Netherlands

**Keywords:** day respiration, Kok method, photorespiration, photosystem II efficiency, reassimilation, Yin method

## Abstract

- The Kok effect refers to the abrupt decrease around the light compensation point in the slope of net photosynthetic rate vs irradiance. Arguably, this switch arises from light inhibition of respiration, allowing the Kok method to estimate day respiration (*R*_d_). Recent analysis suggests that increasing proportions of photorespiration (quantified as Γ*/*C*_c_, the ratio of CO_2_ compensation point Γ* to chloroplast CO_2_ concentration, *C*_c_) with irradiance explain much of the Kok effect. Also, the Kok method has been modified to account for the decrease in PSII photochemical efficiency (Φ_2_) with irradiance.
- Using a model that illustrates how varying *R*_d_, Γ*/*C*_c_, Φ_2_ and proportions of alternative electron transport could engender the Kok effect, we quantified the contribution of these parameters to the Kok effect measured in sunflower across various O_2_ and CO_2_ concentrations and various temperatures.
- Overall, the decreasing Φ_2_ with irradiance explained *c*. 12%, and the varying Γ*/*C*_c_ explained *c*. 25%, of the Kok effect. Maximum real light inhibition of *R*_d_ was much lower than the inhibition derived from the Kok method, but still increased with photorespiration.
- Photorespiration had a dual contribution to the Kok effect, one via the varying Γ*/*C*_c_ and the other via its participation in light inhibition of *R*_d_.

## Introduction

**Fig. 1:**
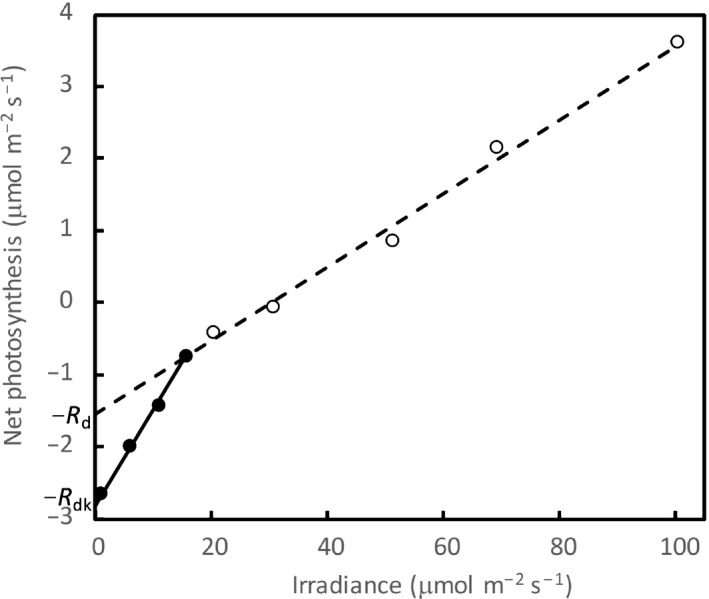
Illustration of a two‐phase photosynthetic response to low irradiances – the Kok effect. The early interpretation of this effect, as suppressing respiration by light, gave rise to the Kok method to estimate respiration in the light (or ‘day respiration’, *R*_d_, the intercept of phase 2; open symbols with the dashed line), which is lower than respiration in the dark (*R*_dk_), the intercept of phase 1 (closed symbols with the solid line).

The Kok effect refers to the abrupt change in the slope of the linear relationship between net photosynthetic rate and irradiance that occurs at very low irradiances, as observed initially in unicellular algae (Kok, 1948, 1949; Healey & Myers, 1971). The switch is reported later in leaves of many higher plant species (e.g. Ishii & Schmid, 1981; Sharp *et al*., 1984; Villar *et al*., 1994; Buckley *et al*., 2017). The slope decreases from the initial higher value to a lower value, mostly at an irradiance value around the light compensation point. This switch has been interpreted as a consequence of light inhibition of respiration, allowing the so‐called Kok method to estimate respiration in the light, or day respiration (*R*_d_), and quantum yield of CO_2_ assimilation (Φ_CO2_) (see Supporting Information Table S1 for all symbol definitions), using the part of the relationship with the lower slope. The absolute value of the estimated *R*_d_ is lower than the respiration in the dark (*R*_dk_) (Fig. 1). The cost of total respiratory activities accounts for *c*. 40% of gross photosynthetic productivity of whole plants (Gifford, 1995; Amthor, 2010). Light inhibition of respiratory activities also occurs at a stand scale (Gong *et al*., 2017), suggesting that it is a general phenomenon, and thus would have a significant impact on projecting the net ecosystem carbon fluxes in biomes across the globe (Heskel *et al*., 2013). For this reason, understanding the Kok effect and its related light inhibition of respiration has continuously received attention (Tcherkez *et al*., 2017a,b).

The lower estimates of *R*_d_ by the Kok method, relative to *R*_dk_, have been confirmed by other gas exchange‐based methods such as the popular Laisk method (Laisk, 1977). By applying the Laisk method to different light intensities, it has been shown that *R*_d_ was progressively inhibited by increasing irradiance (Brooks & Farquhar, 1985; Villar *et al*., 1995). However, this light inhibition has been challenged by the direct measurement of *R*_d_, which exploits the differences in the time course of labelling by carbon isotopes of photosynthetic, photorespiratory and respiratory pathways. For example, using such techniques, Pinelli & Loreto (2003) suggested a significant refixation of respired and photorespired CO_2_ and Loreto *et al*. (2001) calculated that there would be no significant difference between *R*_d_ and *R*_dk_ if the refixation of CO_2_ released from respiration during illumination were taken into account. Similarly, a recent report using a direct method based on isotopic disequilibrium (Gong *et al*., 2018) showed that *R*_d_ was underestimated by the Laisk method. Owing to inconsistent reports of this kind, whether the Kok effect was a result of light inhibition of leaf respiration has been under debate over years.

In fact, according to an extended form of the widely used model of Farquhar *et al*. (1980) for describing the electron transport‐limited photosynthesis, several other mechanisms could also explain the Kok effect. The extended model expresses the net CO_2_ assimilation rate (*A*) as a function of the photosynthetically absorbed irradiance (*I*_abs_) as (Yin *et al*., 2004, 2006):

(Eqn 1) $$A=\frac{1-\Gamma_{*}/C_{c}}{4\left(1+2\Gamma_{*}/C_{c}\right)}f_{\text{aet}}\left(\Phi_{2}\rho_{2}I_{\text{abs}}\right)-R_{d}$$

where *C*_c_ is the CO_2_ concentration at the carboxylating sites of Rubisco, Γ* is the CO_2_ compensation point in the absence of *R*_d_, Φ_2_ is the photochemical efficiency of photosystem II (PSII) electron transport, *ρ*_2_ is the fraction of the absorbed photons partitioned to PSII, and *f*_aet_ is the factor accounting for fractions of alternative electron transport. So, the term (Φ_2_ *ρ*_2_ *I*_abs_) is the flux of PSII electron transport. Parameters *f*_aet_ and *ρ*_2_ can be quantified by the extended model as (Yin *et al*., 2006):

(Eqn 2) $$f_{\text{aet}}=1-\frac{f_{\text{pseudo}}}{1-f_{\text{cyc}}}$$

(Eqn 3) $$\rho_{2}=\frac{1-f_{\text{cyc}}}{1-f_{\text{cyc}}+\frac{\Phi_{2}}{\Phi_{1}}}$$

where Φ_1_ is the photochemical efficiency of PSI electron transport, *f*_cyc_ is the fraction of the PSI electron flux that follows the cyclic electron transport around PSI, and *f*_pseudo_ is the fraction of the PSI electron flux that follows the pseudocyclic electron transport (defined as all noncyclic electron‐consuming pathways other than the Calvin cycle or the photorespiratory cycle).

**Fig. 2:**
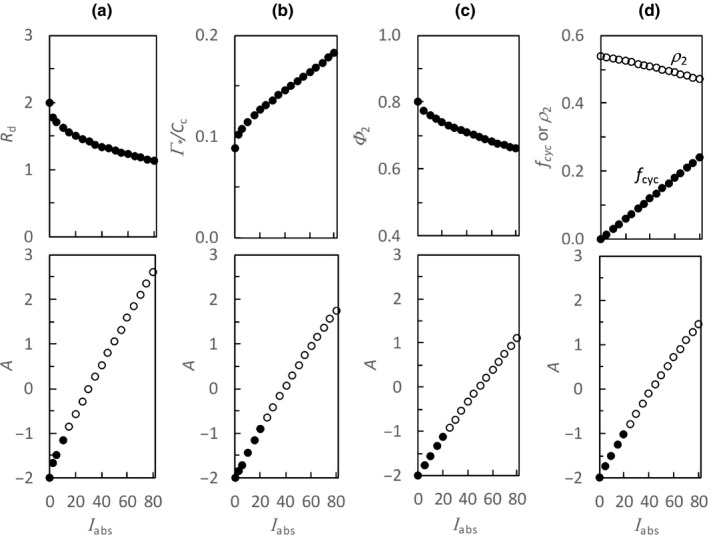
Illustration of the impact of varying values of four parameters (day respiration, *R*_d_; ratio of CO_2_ compensation point to chloroplast CO_2_ concentration, Γ*/*C*_c_; photosystem II photochemical efficiency, Φ_2_; and fraction for cyclic electron transport, *f*_cyc_) with absorbed irradiance *I*_abs_ (upper panels of a–d, respectively) on the shape of the light response curve of net photosynthesis (*A*, lower panels), where there seems to be a transition from a higher‐slope phase (closed symbols) to a lower‐slope phase (open symbols). Curves in lower panels are generated from Eqns (Eqn 1), (Eqn 2), (Eqn 3), in which, when showing the impact of one parameter, other parameters were kept constant. Units are as follows: *I*_abs_, µmol m^−2^ s^−1^; *R*_d_ and *A*, µmol m^−2^ s^−1^; Φ_2_, mol mol^−1^; Γ*/*C*_c_, unitless; and *f*_cyc_ and excitation partitioning to PSII *ρ*_2_, fractions.

Eqn (1) suggests that changes not only in *R*_d_ (Fig. 2a), but also in Γ*/*C*_c_, Φ_2_,* f*_aet_ and *ρ*_2_, with increasing *I*_abs_, result in changes in the slope of *A* vs *I*_abs_. Notably, Farquhar & Busch (2017) recently demonstrated that as a result of regulation of stomatal conductance (*g*_s_) and mesophyll conductance (*g*_m_), *C*_c_ decreased (thus Γ*/*C*_c_ increased) sharply with increasing *I*_abs_ within the range of low irradiances, and that this phenomenon accounted for much of the observed Kok effect (Fig. 2b). A similar argument might be applied to Φ_2_ (Fig. 2c), as Φ_2_ is not constant but decreases with increasing *I*_abs_ (Genty & Harbinson, 1996), even within the range of low irradiances within which the Kok method is used to estimate *R*_d_ and Φ_CO2_ (Yin *et al*., 2011a, 2014). Accounting for the decrease of Φ_2_ with increasing irradiance has resulted in a modified method to estimate *R*_d_ (Yin *et al*., 2009, 2011a). The analysis using the modified method, now known as the Yin method (Tcherkez *et al*., 2017b), indicates that the inhibition of *R*_d_ by light is less than the original Kok method suggests (Yin *et al*., 2011a).

Less information is available on the change in *f*_aet_ or in *ρ*_2_ with *I*_abs_ that could partly explain the Kok effect. Peltier & Sarrey (1988) indicated that the inhibition of chlororespiration (a process in chloroplasts that involves a respiratory electron transport chain within the thylakoid membrane) might be responsible for the Kok effect. Data of Zhang *et al*. (2018) and Ver Sagun *et al*. (2019) suggested that cyclic electron transport around PSI increased with increasing *I*_abs_. If this also applies to the limiting light conditions, an increase in *f*_cyc_ with increasing *I*_abs_ would predict a part of the Kok effect (Fig. 2d). According to Eqns 2 and 3, *f*_cyc_ has a dual effect on the expression of the Kok effect, that is, via both terms *f*_aet_ and *ρ*_2_. Eqn 3 suggests that parameter *ρ*_2_, related to state transition, could be affected not only by *f*_cyc_ (Fig. 2d) but also by the Φ_2_/Φ_1_ ratio. Tcherkez *et al*. (2017a) speculated the possible role of state transition in the Kok effect. The model of Eqns (Eqn 1), (Eqn 2), (Eqn 3) predicts that an increase in *f*_cyc_ or in Φ_2_/Φ_1_ with increasing *I*_abs_ leads to the state transition in favour of PSI with increasing irradiance, and this could engender part of the Kok effect (Fig. 2d).

The Kok effect is not ubiquitous. Early reports found little Kok effect at low O_2_ conditions and in C_4_ plants (Cornic & Jarvis, 1978; Ishii & Murata, 1978). These observations have led to suggestions that photorespiration might be involved in the Kok effect, as confirmed by other studies where photorespiration was manipulated by changing measurement temperatures (Ishii & Schmid, 1981; Way *et al*., 2019) or by lowering leaf water potential (Sharp *et al*., 1984). Again, the model analyses of Farquhar & Busch (2017) demonstrated that the change in Γ*/*C*_c_, therefore, in relative amounts of photorespiration, with increasing *I*_abs_ can explain much of the diminution of the Kok effect in C_4_ plants and at low O_2_ or high CO_2_ concentrations or low temperatures. They also showed that the change in Γ*/*C*_c_ can generate the apparent inhibition of *R*_d_ as inferred by the Laisk method, which is based on *A*–*C*_i_ curves at two or more irradiances (where *C*_i_ is the intercellular CO_2_ concentration). The decrease in *C*_i_ with increasing *I*_abs_ is the result of stomatal regulation, and its influence on estimates of *R*_d_ by the Kok method was noted by Kirschbaum & Farquhar (1987), who proposed a method to correct for this decrease in *C*_i_. The further drawdown in *C*_c_, relative to *C*_i_, is regulated by *g*_m_ (Evans & von Caemmerer, 1996). The Kok method would underestimate *R*_d_ if light‐dependent changes in *C*_i_ (Villar *et al*., 1994) or in *C*_c_ (Ayub *et al*., 2011) are not corrected for. Simple *g*_s_ and *g*_m_ models when coupled with photosynthesis models like Eqns (Eqn 1), (Eqn 2), (Eqn 3) can account for the refixation of CO_2_ released from respiration and photorespiration (von Caemmerer, 2013), and in fact, the refixation fractions of (photo)respired CO_2_ can be calculated analytically from stomatal, mesophyll and carboxylation resistances (Yin & Struik, 2017). As such, the light inhibition of *R*_d_ predicted for photorespiratory conditions by Farquhar & Busch (2017) and the need to correct for light‐dependent changes in *C*_i_ and *C*_c_ are basically analogous to the statement of Loreto *et al*. (2001) that the lower *R*_d_ than *R*_dk_ resulted from the failure of the original Kok or Laisk methods in accounting for the refixation of respired CO_2_ in the light.

However, there are cases where the Kok effect is not always associated with photorespiration. The change in the slope was occasionally observed to be present under high‐CO_2_ conditions (Sharp *et al*., 1984), and in C_4_ leaves and under low‐O_2_ conditions albeit to a smaller extent (Yin *et al*., 2011a). Gong *et al*. (2015) reported an even lower *R*_d_ : *R*_dk_ ratio in C_4_ than in C_3_ leaves. Buckley *et al*. (2017) observed a similar extent of change in the slope under both 21% and 2% O_2_ conditions for broadbean (*Vicia faba*) mature leaves. Nevertheless, the Kok effects reported in the early years (Kok, 1949; Ishii & Schmid, 1981; Sharp *et al*., 1984) are mostly associated with the abrupt transition in the slope (Fig. 1), whereas the *g*_s_–*g*_m_ photosynthesis model predicts only a smooth transition (Farquhar & Busch, 2017).

Of the possible mechanisms (*R*_d_, Γ*/*C*_c_, Φ_2_, *f*_aet_ and/or *ρ*_2_) highlighted by Eqns (Eqn 1), (Eqn 2), (Eqn 3) that potentially explain the magnitude of the Kok effect (Fig. 2), *f*_aet_ and *ρ*_2_ are hard to measure accurately by existing equipment, especially at low irradiances along the Kok curve. Also, the pattern of changing *R*_d_ in response to *I*_abs_ is hard to quantify with existing methods. In this study, we will illustrate, using Eqns (Eqn 1), (Eqn 2), (Eqn 3), that how *R*_d_ responds to *I*_abs_ would have relevance to the Kok effect and in estimating Φ_CO2_. We surmise that if the varying Γ*/*C*_c_ ratio is a major factor accounting for the Kok effect, as stated by Farquhar & Busch (2017), then the magnitude of the Kok effect should be associated with the Γ*/*C*_c_ ratio, regardless of how the variation of this ratio is created. To this end, we designed an experiment in which we used various O_2_ and CO_2_ concentrations or temperatures to generate varying relative amounts of photorespiration, that is, various Γ*/*C*_c_ ratios. Based on a modelling analysis of the experimental data we quantitatively assess: whether the change of Γ*/*C*_c_ and the decrease of Φ_2_ with increasing *I*_abs_ could explain, in part, the Kok effect; if so, what the relative contribution of the two components is in determining the Kok effect; and what the maximum real inhibition of *R*_d_ by light is. We demonstrate that our results help to identify common threads explaining seemingly contradictory findings among previous studies on *R*_d_.

## Materials and Methods

### Plant materials and measurements

Plants of sunflower (*Helianthus annuus*, cv ‘Sunspot’) were grown in pots in a growth chamber (day : night temperature, 25 : 20°C; relative humidity, 70%; photon flux density, *c*. 500 μmol m^−2^ s^−1^ at the soil level; photoperiod, 16 h, 06:00–22:00 h) in Wageningen. Five seeds were sown and seedlings were thinned to one plant per 7 l pot. Initial amounts of soil nitrogen (N), phosphorus (P) and potassium (K) were 0.62, 0.83, and 1.04 g per pot, respectively. Nutrient solution was added two or three times per week based on the expected plant growth. Seeds were sown weekly for 4 wk, creating four replications. Measurements were conducted on the 11^th^ or 12^th^ fully expanded leaf counting from the bottom, using one plant per replication.

An open gas exchange system (Li‐Cor 6800; Li‐Cor Inc., Lincoln, NE, USA) and an integrated fluorescence chamber head of 6 cm^2^ were used for three sets of measurements, in which various O_2_ or CO_2_ concentrations or various temperatures were used to create different amounts of photorespiration (Table 1). The first set used five O_2_ concentrations. Four cylinders containing different mixtures of O_2_ and N_2_ were used. Gas from the cylinder was supplied to the Li‐Cor 6800 where CO_2_ was blended with O_2_. For the second set, five different ambient CO_2_ (*C*_a_) concentrations in the leaf chamber were used (Table 1). For the third set, four leaf temperatures were used (Table 1). A flow rate of 200 μmol s^−1^ was used, and leaf‐to‐air vapour pressure difference was maintained within 0.8–1.6 kPa, for all measurements.

**Table 1:** Levels of O_2_, ambient CO_2_ and leaf temperature in three sets of measurements on sunflower leaves.

Set	O_2_ (%)	CO_2_ (µmol mol^−1^)	Temperature (°C)
1	2, 10, 21, 35, 50	400	25
2	21	100, 250, 400, 550, 700	25
3	21	400	15, 25, 30, 35

For a given O_2_, CO_2_ or temperature, a photosynthetic response curve to incident irradiance (*A*−*I*_inc_) was measured. Leaves were first acclimated under 80 μmol m^−2^ s^−1^ until *A* reached a steady state, which took *c*. 45 min. Measurements were then undertaken using a sequence of 80, 70, 60, 50, 40, 30, 25, 20, 15, 10, and 5 μmol m^−2^ s^−1^, with 6 min for each step. For measurements in each of the first two sets, O_2_ or CO_2_ concentrations were chosen randomly. Measurements of the temperature set were conducted after the O_2_ and CO_2_ sets to avoid possible after‐effects of high temperature on leaves. For the same reason, the four temperatures were set up from low to high rather than randomly.

After the measurements for *A*−*I*_inc_ curves, *A*−*C*_i_ curves were determined to provide extra data to estimate *g*_m_. Leaves were adapted to an *I*_inc_ of 100 μmol m^−2^ s^−1^ at 25°C and 21% O_2_ until *A* became stable, and curves were measured using a *C*_a_ sequence of 400, 200, 100, 75, 50, 400, 400, 550, 800 and 1500 μmol mol^−1^, with 3 min per step. Apparent *A*–*C*_i_ curves were also assessed with heat‐killed leaves, which showed that CO_2_ leakage was negligible during our measurement using the Li‐Cor 6800.

For each step of either the *A*−*I*_inc_ or *A*−*C*_i_ curve, PSII photochemical operating efficiency (Φ_2_) was determined by Chl fluorescence as (1−*F*_s_/*F*_m_′), where *F*_s_ is the steady‐state fluorescence and *F*_m_′ is the maximum fluorescence as revealed using the single flash of *c*. 8500 µmol m^−2^ s^−1^ for a duration of 1.0 s. We did not use the multiphase method to determine *F*_m_′ because all measurements were undertaken at low irradiances.

Leaf spots used for measurements were punched out, and leaf discs were measured for light absorption (STS‐VIS miniature spectrometer; Ocean Optics, Dunedin, FL, USA), twice per disc, to represent average absorption at this spot. After measuring their areas, leaf discs were dried in a 70*°*C oven for 24 h to determine dry matter. Each dry leaf disc was then ground into powder, and samples of 1–3 mg were analysed for N concentrations with an EA1108 CHN‐O Element Analyzer (Fisons Instruments, Waltham, MA, USA) using the Dumas combustion method.

### Data and modelling analyses

All the leaf spots had a similar N content. The average leaf N was 1.6 g m^−2^ and average leaf absorptance was 85%. Variation among replications was small, and replicate average values were used for analysis.

Data of *A* vs *I*_abs_ were inspected to identify the irradiance at the Kok transition point (*I*_abs,t_), based on the highest average *r* ^2^ of linear regression on points both below and above the candidate *I*_abs,t_ of each curve. The regression slopes below and above *I*_abs,t_ were denoted as *b*_1_ and *b*_2_, respectively, and the *b*_1_ : *b*_2_ ratio was calculated. The intercept of the regression after *I*_abs,t_ is the day respiration estimated by the Kok method. Here, the intercepts of regression lines before and after *I*_abs,t_ are denoted as *r*_d1_ and *r*_d2_, respectively. According to the original interpretation of the Kok effect (Fig. 1), *r*_d1_ is equivalent to the respiration rate in the darkness, *R*_dk_.

To examine if the decrease of Φ_2_ with increasing *I*_abs_ could partly explain the Kok effect, plots of *A* vs *I*_abs_ Φ_2_ were made. To be compared with the *A*−*I*_abs_ plots, data points were allocated according to *I*_abs,t_ identified earlier, and linear regression slopes both below and above *I*_abs,t_ were denoted as *B*_1_ and *B*_2_, respectively. Any decrease in the *B*_1_ : *B*_2_ ratio, relative to the *b*_1_ : *b*_2_ ratio, would suggest that the decrease of Φ_2_ with increasing *I*_abs_ could partly explain the Kok effect. The intercept of the linear plot of *A* vs *I*_abs_ Φ_2_/4 after the Kok break point is the day respiration estimated by the Yin method (Yin *et al*., 2009, 2011a). As the intercept remains unchanged if the linear plot is made here for *A* vs *I*_abs_ Φ_2_, the intercepts of *A*−*I*_abs_ Φ_2_ lines before and after *I*_abs,t_ are denoted as *R*_D1_ and *R*_D2_, respectively.

To assess the impact of Γ*/*C*_c_ on the Kok effect, *C*_c_ has to be known. To that end, we estimated *g*_m_ using all data from combined gas exchange and Chl fluorescence measurements. *g*_m_ is known to vary with temperature (Bernacchi *et al*., 2002), but whether *g*_m_ varies with *C*_i_ or with *I*_inc_ or O_2_ is uncertain. Furthermore, recent literature suggests the necessity to dissect mesophyll resistance into its components (Tholen *et al*., 2012) and to consider the intracellular arrangements of organelles (Yin & Struik, 2017; Ubierna *et al*., 2019; Yin *et al*., 2020). Here we consider three *g*_m_ modes: mode i assumes that *g*_m_ varies only with temperature, but not with either *C*_i_ or *I*_inc_ or O_2_; mode ii assumes that *g*_m_ varies with all these factors; and mode iii is similar to mode ii but uses an additional factor *m* that lumps subresistance proportions and several intracellular properties of mesophyll organelles (Yin *et al*., 2020). For mode i, we estimated *g*_m_ by fitting, the NRH‐A method based on the non‐rectangular hyperbolic equation for CO_2_‐assimilation, described by Yin & Struik (2009) to all data (including *A*−*C*_i_ curves). Like photosynthetic rate, *g*_m_ has generally an optimum response to temperature (e.g. Bernacchi *et al*., 2002; Warren & Dreyer, 2006; but with caution, see von Caemmerer & Evans, 2015), and we assumed that this response followed a normal distribution function, with an optimum temperature of 30°C: *g*_m_ = *g*_m30_ exp{−[(*T*−30)/Ω]^2^}, which has a minimum number of parameters to estimate. We incorporated these relationships into the NRH‐A method to fit parameter Ω. For modes ii and iii, we used an equation described by Yin *et al*. (2009), *g*_m_ = *δ*(*A* + *R*_d_)/(*C*_c _− Γ*), which can semi‐empirically accommodate the response (if observed) of *g*_m_ to *I*_inc_, *C*_i_, O_2_ and temperature. Here, it is the unitless coefficient *δ* that is an explicit parameter to be estimated, and *δ* represents the carboxylation resistance : mesophyll resistance ratio (Yin *et al*., 2020). For each mode, the simultaneously estimated parameters together with *g*_m_ or *δ* were: the calibration factor(s) that converts Chl fluorescence‐based PSII photochemical efficiency (Φ_2_) into linear electron transport rate (*J*), with *J* = *sI*_inc_ Φ_2_ (Yin *et al*., 2009); and Rubisco specificity at 25°C (*S*_c/o25_). The values of *S*_c/o_ for other temperatures were calculated from the relation Γ* = 0.5*O*/*S*_c/o_ (where *O* is the concentration of oxygen; Farquhar *et al*., 1980; von Caemmerer, 2013) and the Arrhenius equation using 24 460 J mol^−1^ of Bernacchi *et al*. (2002) as the activation energy for Γ* (using other activation‐energy estimates (e.g. Walker *et al*., 2013; Yin *et al*., 2014) had little impact on our calculated Γ*/*C*_c_ ratios). In view of the reasoning of Farquhar & Busch (2017), we used *R*_D1_ of each curve as input for the *R*_d_ term of the model in fitting. The fitting procedures for three modes were implemented using the GAUSS method in proc nlin of SAS (SAS Institute Inc, Cary, NC, USA), and the SAS codes can be obtained upon request. The SAS output gave the fitted *A* for each measurement point, with which *C*_c_ was then solved from the model of Farquhar et al. (1980) as: *C*_c_ = Γ* [*J*/4 + 2(*A* + *R*_d_)]/[*J*/4 – (*A* + *R*_d_)].

## Results

### Forms of light inhibition of *R*_d_ in relation to the Kok effect

**Fig. 3:**
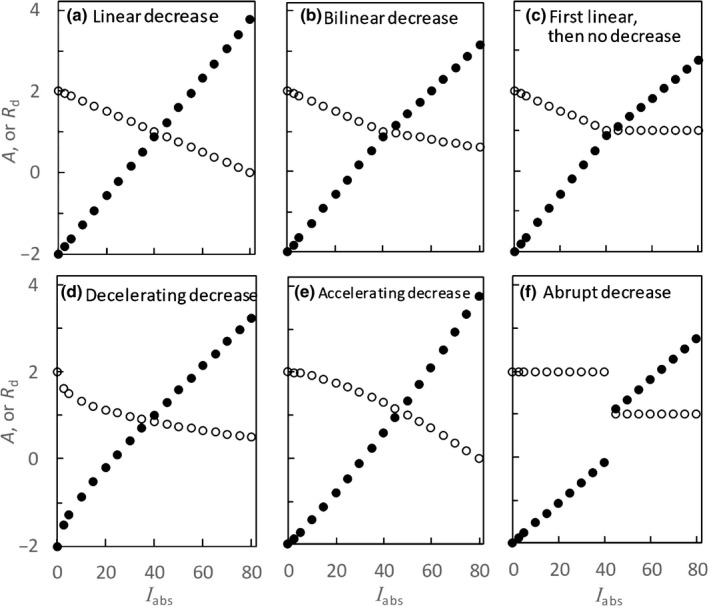
Illustration of six scenarios (a–f) for the so‐called ‘progressive’ decrease of day respiration, *R*_d_, with absorbed irradiance, *I*_abs_ (open circles), and their impact on the shape of the light response curve of net photosynthesis, *A* (closed circles). Units: *I*_abs_, µmol m^−2^ s^−1^; *R*_d_ and *A*, µmol m^−2^ s^−1^.

We consider all possible scenarios in interpreting the often‐said ‘progressive’ inhibition of respiration by light, and examine, based on Eqn 1, the consequence of these scenarios on the shape of *A*−*I*_abs_ curves within a range of the low irradiances (Fig. 3).

The scenario ‘continuously linear decrease’ of *R*_d_ with light (Fig. 3a) did not at all result in a break in the linear relationship. Only two ‘bilinear’ scenarios can generate the Kok effect with an abrupt transition point (Fig. 3b,c). The ‘continuously decelerating decrease’ scenario also generated the Kok effect but without the abrupt break point (Fig. 3d). For an ‘accelerating decrease’ scenario, *R*_d_ was also progressively suppressed by light, but this scenario generated an *A*−*I*_abs_ curve where the slope did not decrease but increased (Fig. 3e), thereby being unable to reproduce the Kok curve. Finally, an ‘abrupt suppression’ scenario cannot be ruled out, but this scenario generated two linear discontinued segments with the same slope (Fig. 3f), thereby being unable to reproduce the Kok effect either.

As illustrated in Fig. 3, the difference in scenarios also has implications for the estimation of Φ_CO2_. Only in the second ‘bilinear’ scenario (Fig. 3c) and the abrupt‐suppression scenario (Fig. 3f) can Φ_CO2_ be reliably estimated by the Kok method as the slope of the *A*−*I*_abs_ curve above the break point. For other scenarios, the slope represents the combined yield of photosynthesis and of the component of light suppression of *R*_d_. In fact, it is the scenario of Fig. 3(c) that the Kok method relies on to estimate *R*_d_ and Φ_CO2_.

### The observed Kok effect across various O_2_ and CO_2_ concentrations and various temperatures

**Fig. 4:**
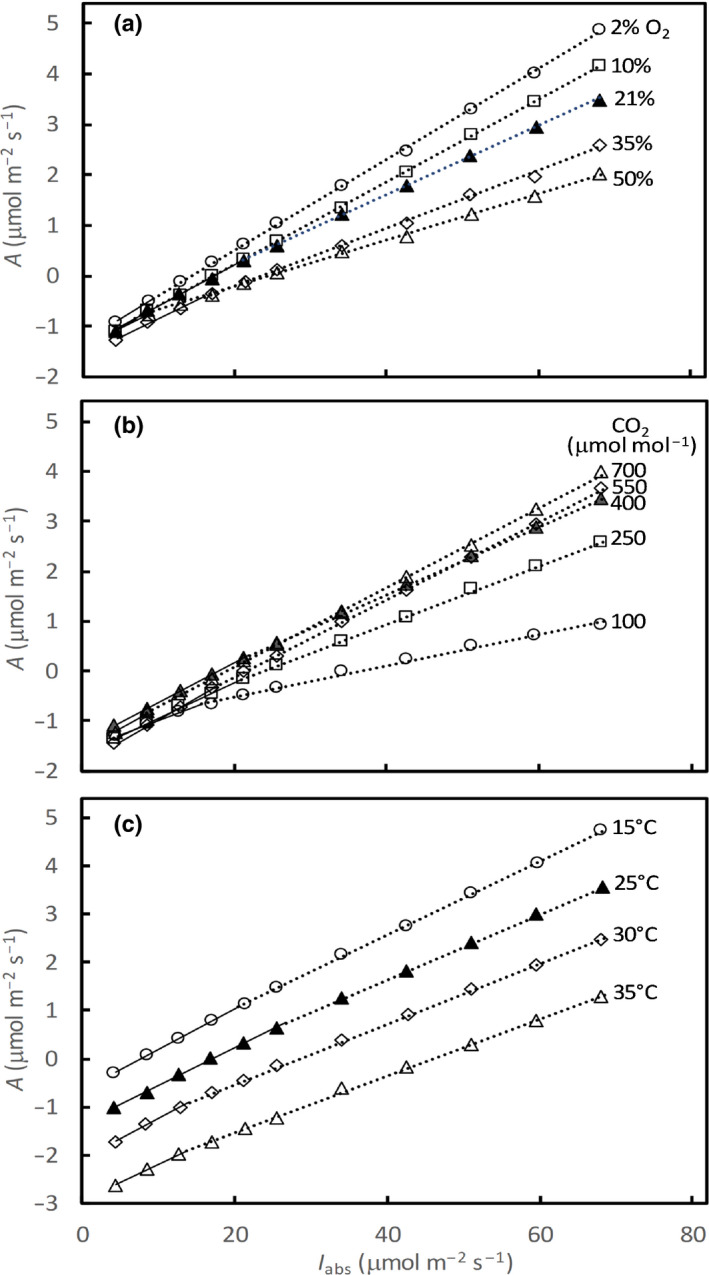
Net photosynthesis rates (*A*) vs absorbed irradiance (*I*_abs_), based on measured data from five O_2_ concentrations (a), five CO_2_ concentrations (b), and four temperatures (c) for sunflower leaves. Points represent the means of measurements on four replicated leaves. Continuous lines are for phase 1, and dotted lines are for phase 2, of the Kok plot, drawn from parameter estimates as given in Tables 2 and 3.

Linear plots of *A* vs *I*_abs_ using our experimental data identified the Kok break point in each curve (Fig. 4). The maximum values of the slope below (phase 1, *b*_1_) and above the break point (phase 2, *b*_2_) were achieved at 2% O_2_, and were 0.095 and 0.090 mol mol^−1^, respectively (Table 2), similar to experimentally measured (Long *et al*., 1993) or theoretically inferred Φ_CO2_ (Yin *et al.*, 2006) for C_3_ species under nonphotorespiratory conditions. A change in the slope from phase 1 to phase 2 became more significant with increasing O_2_ concentrations, with decreasing CO_2_ concentrations, and with increasing temperature (Fig. 4). The *b*_1_ : *b*_2_ ratio increased from 1.06 at 2% O_2_ to 1.69 at 50% O_2_, from 1.07 at 700 µmol mol^−1^ CO_2_ to 1.83 at 100 µmol mol^−1^ CO_2_, and from 1.10 at 15°C to > 1.30 at 30–35°C (Table 2).

**Table 2:** Estimates of the slope values of phase 1 (*b*_1_) and phase 2 (*b*_2_) in the *A* vs *I*_abs_ plot or of the slope values of phase 1 (*B*_1_) and phase 2 (*B*_2_) in the *A* vs *I*_abs_Φ_2_ plot for sunflower leaves.

		*A* vs *I*_abs_ plot			*A* vs *I*_abs_Φ_2_ plot		
		*b*_1_	*b*_2_	*b*_1_ : *b*_2_	*B*_1_	*B*_2_	*B*_1_ : *B*_2_
O_2_ (%)	2	0.095 (0.004)	0.090 (0.001)	1.06	0.118 (0.004)	0.115 (0.001)	1.03
	10	0.083 (0.003)	0.082 (0.001)	1.02	0.109 (0.003)	0.106 (0.001)	1.02
	21	0.081 (0.003)	0.068 (0.001)	1.19	0.103 (0.003)	0.090 (0.001)	1.15
	35	0.071 (0.005)	0.057 (0.001)	1.24	0.092 (0.006)	0.076 (0.001)	1.21
	50	0.078 (0.011)	0.046 (0.001)	1.69	0.105 (0.014)	0.063 (0.001)	1.67
CO_2_ (µmol mol^−1^)	100	0.057 (0.008)	0.031 (0.001)	1.83	0.073 (0.005)	0.042 (0.001)	1.74
	250	0.070 (0.003)	0.058 (0.001)	1.21	0.088 (0.002)	0.075 (0.001)	1.17
	400	0.080 (0.001)	0.068 (0.000)	1.18	0.099 (0.001)	0.086 (0.000)	1.15
	550	0.086 (0.003)	0.078 (0.001)	1.11	0.108 (0.003)	0.101 (0.001)	1.07
	700	0.085 (0.003)	0.080 (0.001)	1.07	0.102 (0.003)	0.103 (0.001)	0.99
Temperature (°C)	15	0.085 (0.002)	0.077 (0.000)	1.10	0.105 (0.002)	0.098 (0.000)	1.06
	25	0.079 (0.001)	0.068 (0.001)	1.16	0.099 (0.001)	0.088 (0.001)	1.13
	30	0.086 (0.004)	0.063 (0.001)	1.37	0.112 (0.004)	0.085 (0.001)	1.32
	35	0.078 (0.006)	0.059 (0.001)	1.33	0.105 (0.006)	0.081 (0.001)	1.29

The slope values have a unit of mol mol^−1^ and standard errors of the estimates are given in brackets; data used for estimation were from the three sets of measurements as described in Table 1.

*A*, net rate of leaf photosynthesis (μmol m^−2^ s^−1^); *I*_abs_, irradiance absorbed by leaf photosynthetic pigments (μmol m^−2^ s^−1^); Φ_2_, photochemical efficiency of photosystem II electron transport (mol mol^−1^).

Similarly, the difference in the estimated respiration for phase 1 and phase 2, denoted as *r*_d1_ and *r*_d2_, respectively, became more significant with increasing O_2_ concentrations, decreasing CO_2_ concentrations, and increasing temperature (Table 3). With the estimated *b*_1_, *b*_2_, *r*_d1_ and *r*_d2_, the irradiance for the Kok break point, *I*_abs,t_, can be calculated, and it varied from 7 to 27 µmol m^−2^ s^−1^ (Table 3).

**Table 3:** Estimates of the intercept values of phase 1 (*r*_d1_) and phase 2 (*r*_d2_) in the *A* vs *I*_abs_ plot or of the intercept values of phase 1 (*R*_D1_) and phase 2 (*R*_D2_) in the *A* vs *I*_abs_ Φ_2_ plot for sunflower leaves.

		*A* vs *I*_abs_ plot			*A* vs *I*_abs_Φ_2_ plot			*I*_abs,t_
		*r*_d1_	*r*_d2_	*r*_d1_ : *r*_d2_	*R*_D1_	*R*_D2_	*R _D1_ : R _D2_*	
O_2_ (%)	2	1.33 (0.04)	1.28 (0.04)	1.04	1.34 (0.03)	1.34 (0.03)	1.00	9.1
	10	1.42 (0.03)	1.41 (0.03)	1.01	1.46 (0.03)	1.46 (0.03)	1.00	6.8
	21	1.41 (0.03)	1.14 (0.03)	1.24	1.41 (0.03)	1.21 (0.02)	1.17	21.0
	35	1.55 (0.05)	1.33 (0.05)	1.17	1.56 (0.06)	1.38 (0.05)	1.13	16.0
	50	1.43 (0.07)	1.13 (0.02)	1.26	1.44 (0.07)	1.15 (0.02)	1.25	9.3
CO_2_ (µmol mol^−1^)	100	1.55 (0.07)	1.14 (0.04)	1.35	1.55 (0.05)	1.17 (0.03)	1.32	15.6
	250	1.64 (0.03)	1.39 (0.03)	1.18	1.64 (0.02)	1.43 (0.02)	1.15	20.9
	400	1.44 (0.01)	1.16 (0.02)	1.24	1.44 (0.01)	1.21 (0.01)	1.19	21.9
	550	1.83 (0.03)	1.68 (0.03)	1.09	1.83 (0.03)	1.73 (0.03)	1.06	17.4
	700	1.57 (0.04)	1.53 (0.04)	1.03	1.54 (0.04)	1.56 (0.04)	0.99	8.5
Temperature (°C)	15	0.66 (0.02)	0.50 (0.02)	1.32	0.67 (0.01)	0.56 (0.01)	1.18	20.4
	25	1.36 (0.02)	1.06 (0.03)	1.28	1.36 (0.01)	1.12 (0.02)	1.22	26.7
	30	2.10 (0.04)	1.78 (0.02)	1.18	2.10 (0.03)	1.82 (0.02)	1.15	14.0
	35	2.98 (0.06)	2.71 (0.03)	1.10	2.98 (0.06)	2.75 (0.03)	1.08	13.6

The intercept values have a unit of μmol m^−2^ s^−1^ and standard errors of the estimates are given in brackets; data used for estimation were from the three sets of measurements as described in Table 1.

*A*, net rate of leaf photosynthesis (μmol m^−2^ s^−1^); *I*_abs_, irradiance absorbed by leaf photosynthetic pigments (μmol m^−2^ s^−1^); Φ_2_, photochemical efficiency of photosystem II electron transport (mol mol^−1^); *I*_abs,t_, the calculated value of *I*_abs_ (μmol m^−2^ s^−1^) for the transition from phase 1 to phase 2 from the *A* vs *I*_abs_ plot.

### The variable Φ_2_ as a possible cause for the Kok effect

As with previous reports (Yin *et al*., 2011a, 2014), Φ_2_ decreased with increasing irradiances in all three sets of measurements (Fig. S1). Compared with the *A* vs *I*_abs_ plots, the *A* vs *I*_abs_ Φ_2_ plots had a similar shape (thus, they are not shown), but the obtained *B*_1_ : *B*_2_ ratios were slightly lower than the *b*_1_ : *b*_2_ ratios (Table 2). As expected, the regression of *A* against *I*_abs_ Φ_2_ yielded consistently lower intercepts, and therefore higher estimates, *R*_D1_ and *R*_D2_, compared with *r*_d1_ and *r*_d2_, respectively, confirming the results of earlier studies (Yin *et al*., 2011a). For the same reason, the *R*_D1_ : *R*_D2_ ratios were smaller than the *r*_d1_ : *r*_d2_ ratios (Table 3). There were no consistent trends for absolute values of *r*_d1_, *r*_d2_, *R*_D1_ and *R*_D2_ with changing O_2_ or CO_2_ concentrations; but unsurprisingly they increased consistently with increasing temperature (Table 3).

### Association of the Kok effect with the variable Γ*/C_c_

We estimated parameter values of the aforementioned three *g*_m_ modes. The estimate of the *m* factor for mode iii was 0, which means that modes ii and iii had identical results. To test whether a nonzero *m* factor influenced the calculated *C*_c_, we fixed *m* to 0.3, our recent estimate of this parameter (Yin *et al*., 2020). The three *g*_m_ modes yielded the same goodness of fit with *R* ^2^ of 0.966 (Table S2). The modelled *A* by the three modes did not differ essentially (Fig. S2a). Using the modelled *A*, we calculated the Γ*/*C*_c_ ratio across irradiance of all the three sets of measurements. The Γ*/*C*_c_ ratios calculated by mode ii or iii at first very low irradiances were more variable than those given by mode i (results not shown), but the average Γ*/*C*_c_ ratio along a given *A*−*I*_inc_ curve did not differ much between the three modes (Fig. S2b). We also used the variable J method of Harley *et al* (1992) to inspect any variation of *g*_m_ and found no evidence that *g*_m_ varied with either *C*_i_ or with *I*_inc_ or with O_2_. In the following analysis, we show the results based on the estimate using mode i, as they did not differ much from those using mode ii or iii.

**Fig. 5:**
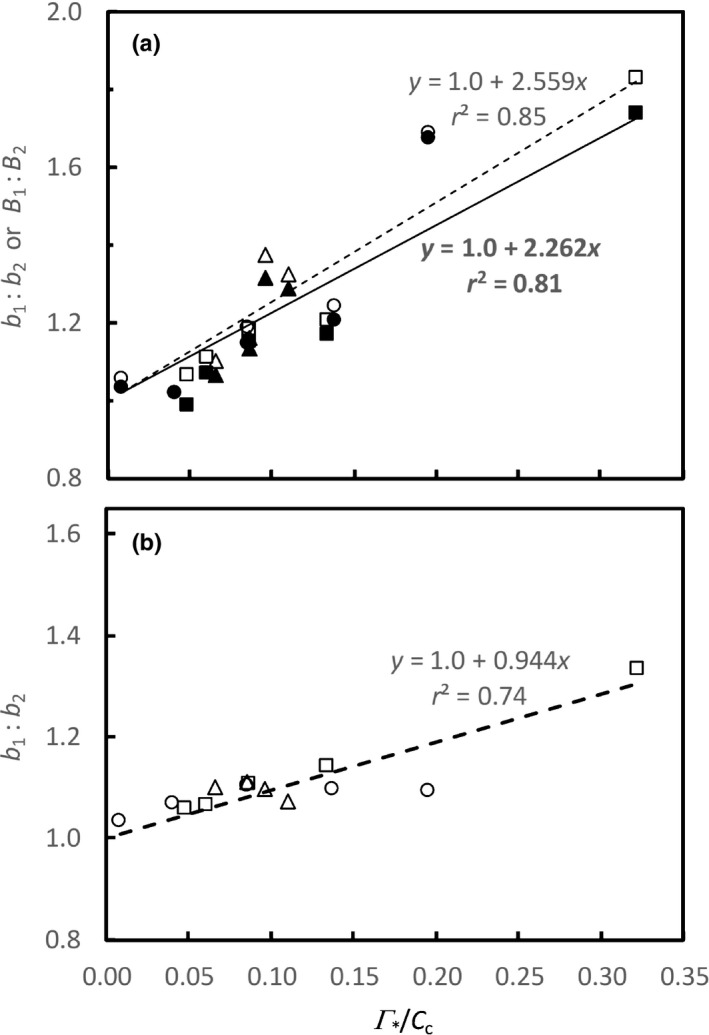
The slope ratios of phase 1 to phase 2 in the net photosynthesis rate (*A*) vs absorbed irradiance (*I*_abs_) plot (i.e. *b*_1_ : *b*_2_ ratios; open symbols) or in the *A* vs *I*_abs_Φ_2_ plot (i.e. *B*_1_ : *B*_2_ ratios; closed symbols) based on measured values of *A* (a), or the *b*_1_ : *b*_2_ ratios based on modelled values of *A* (b), plotted against ratios of CO_2_ compensation point to chloroplast CO_2_ concentration (Γ*/*C*_c_) across various O_2_ concentrations (circles), CO_2_ concentrations (squares) and temperatures (triangles) for sunflower leaves. Equations represent the regression lines that pass the (0, 1) point. Φ_2_, photosystem II photochemical efficiency.

The obtained average Γ*/*C*_c_ ratio varied from 0.008 to 0.195 when O_2_ varied from 2% to 50%, from 0.322 to 0.049 when CO_2_ varied from 100 to 700 µmol mol^−1^, and from 0.066 to 0.110 when temperature varied from 15 to 35°C. Plotting the *b*_1_ : *b*_2_ ratio or the *B*_1_ : *B*_2_ ratio against the Γ*/*C*_c_ ratio showed linear relationships, and because these linear relations did not differ significantly among the three sets of measurements, the common regression line was obtained, and the intercept of the line at the zero Γ*/*C*_c_ ratio was close to 1 (Fig. 5a).

### The extents to which the Kok effect was explained by variable Φ_2_ and Γ*/C_c_

The strong correlation of the *b*_1_ : *b*_2_ or *B*_1_ : *B*_2_ ratio with the Γ*/*C*_c_ ratio (with *R* ^2^ > 0.80; Fig. 5a) does not mean that the varying Γ*/*C*_c_ ratio can explain more than 80% of the Kok effect because other factors (such as *R*_d_ and *f*_aet_) may vary with Γ*/*C*_c_ as well. However, the relative difference in the slope value between the *b*_1_ : *b*_2_ vs the Γ*/*C*_c_ plot (2.559, Fig. 5a) and the *B*_1_ : *B*_2_ vs the Γ*/*C*_c_ plot (2.262; Fig. 5a) should quantify the contribution of the decreasing Φ_2_ in explaining the Kok effect. This relative difference was (2.559–2.226)/2.559 × 100% = 11.6%, suggesting that, overall, the varying Φ_2_ explained *c*. 12% of the Kok effect across varying O_2_ and CO_2_ concentrations and varying temperatures.

We plotted the modelled *A* against *I*_abs_ to generate slope values of *b*_1_ and *b*_2_, and thereby the modelled *b*_1_ : *b*_2_ ratios. The modelled *b*_1_ : *b*_2_ ratios did increase with the Γ*/*C*_c_ ratio (Fig. 5b), in line with the statement of Farquhar & Busch (2017) that the changing Γ*/*C*_c_ ratio explains much of the observed Kok effect. Farquhar & Busch (2017) did not estimate quantitatively the extent of the explanation.

The modelled *b*_1_ : *b*_2_ ratios were lower than the observed *b*_1_ : *b*_2_ ratios shown in Table 2. Our prediction used measured *C*_i_ and Φ_2_ as input and took the effect of *g*_m_ and Γ* into account, and therefore the effects of varying Φ_2_ and Γ*/*C*_c_ were already considered in the modelling. This suggests that the plot of the modelled *b*_1_ : *b*_2_ ratios vs the Γ*/*C*_c_ ratios should reflect the combined effect of both varying Φ_2_ and varying Γ*/*C*_c_. The intercept of the plot for the modelled *b*_1_ : *b*_2_ ratios vs the Γ*/*C*_c_ ratios was again close to 1; but its slope was 0.944 (Fig. 5b), much lower than 2.559 – the slope of the observed *b*_1_ : *b*_2_ ratios vs the Γ*/*C*_c_ ratios (Fig. 5a). As the intercept remained unaltered, this indicates that the combined contribution of varying Φ_2_ and Γ*/*C*_c_ to the observed Kok effect can be estimated from slope values, that is, *c*. 36.9% (= 0.944/2.559 × 100%). Therefore, the effect of varying Γ*/*C*_c_ alone explained *c*. 25.3% (36.9–11.6%) of the observed Kok effect across various O_2_ and CO_2_ concentrations and various temperatures.

### Quantifying the maximum extent of inhibition of day respiration by light

Our modelling procedure aimed to quantify the contribution of Φ_2_ and varying Γ*/*C*_c_, and therefore, as is the usual case, assumed that *R*_d_ and *f*_aet_ did not vary with irradiance or with measurement O_2_ and CO_2_ conditions. The remaining unexplained contributions (*c*. 63%) must be a result of light inhibition of *R*_d_ and possibly variable *f*_aet_ and/or *ρ*_2_. We are not able to separate the contribution of light inhibition of *R*_d_ from the effect of variable *f*_aet_ and/or *ρ*_2_ if the variation of *f*_aet_ and/or *ρ*_2_ with irradiance cannot be ruled out. If we assume that the variation of either *f*_aet_ and/or *ρ*_2_ with irradiance is negligible with the limiting light range, as is often assumed in measuring Φ_CO2_, we can quantify the real inhibition of *R*_d_ by light by removing the effect of changing Φ_2_ and Γ*/*C*_c_, as described in the following. As such, this estimate should be considered as the maximum real inhibition of *R*_d_ by light.

The apparent relative inhibition in case of the Yin method is:

(Eqn 4) $${\text{Inhibition}}_{\text{apparent}}\left(\%\right)=\frac{R_{\text{D1measured}}-R_{\text{D2measured}}}{R_{\text{D1measured}}}\times 100$$

**Fig. 6:**
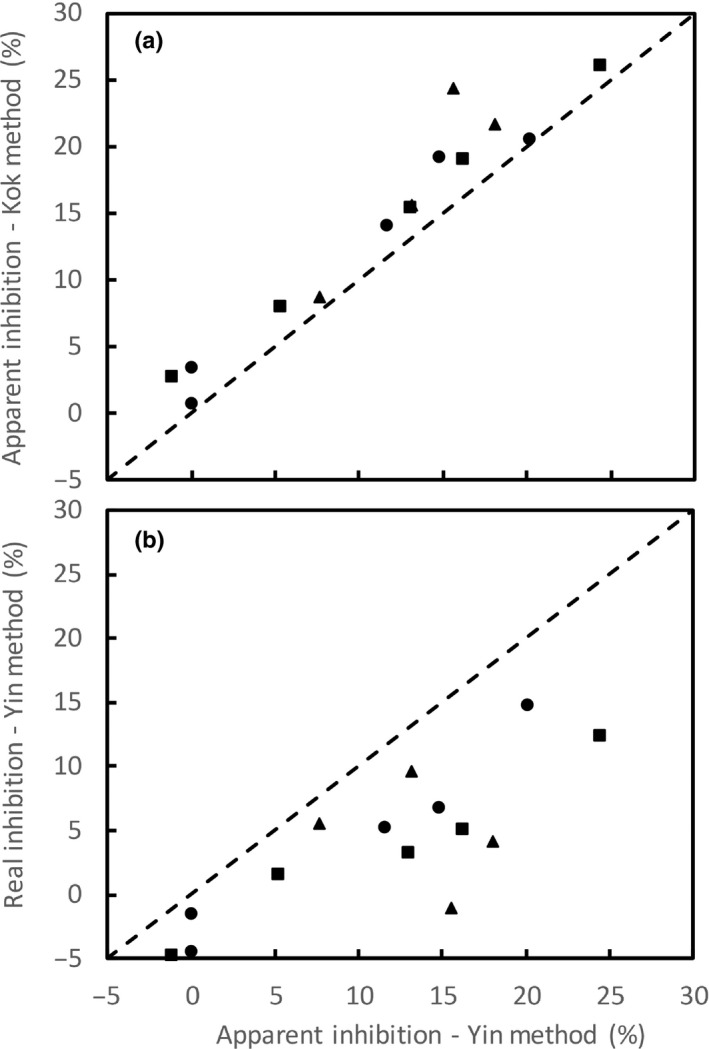
Relative apparent light inhibition of day respiration, *R*_d_, identified by the Kok method vs that identified by the Yin method (a), and relative real light inhibition vs the relative apparent light inhibition of *R*_d_ both identified by the Yin method (b), across various O_2_ concentrations (circles), CO_2_ concentrations (squares) and temperatures (triangles) for sunflower leaves. The dashed diagonal represents the 1 : 1 line, at which *y* = *x*.

The similar apparent relative inhibition can be proposed for the Kok method. The apparent inhibition was higher according to the Kok method than according to the Yin method (Fig. 6a), owing to the fact that the Kok method ignores the decrease of Φ_2_ with irradiance. Overall, the Kok method overestimated the apparent inhibition of *R*_d_ by *c*. 18%, as compared with the Yin method.

Plotting the modelled *A* against *I*_abs_ resulted in lower estimates of *r*_d2_ than *r*_d1_ and plotting the modelled *A* against *I*_abs_ Φ_2_ also resulted in lower estimates of *R*_D2_ than *R*_D1_ than their respective estimates using the observed *A* (results not shown), although a single value of *R*_d_ was used for each curve in modelling. This confirmed the analysis of Farquhar & Busch (2017) that the apparent inhibition of *R*_d_ by light was partly a result of the artefact of changing Γ*/*C*_c_ with irradiance. The real relative inhibition of *R*_d_ by light can be calculated as:

(Eqn 5) $${\text{Inhibition}}_{\text{real}}\left(\%\right)=\frac{\left(R_{\text{D1measured}}-R_{\text{D2measured}}\right)-\left(R_{\text{D1modelled}}-R_{\text{D2modelled}}\right)}{R_{\text{D1measured}}}\times 100$$

**Fig. 7:**
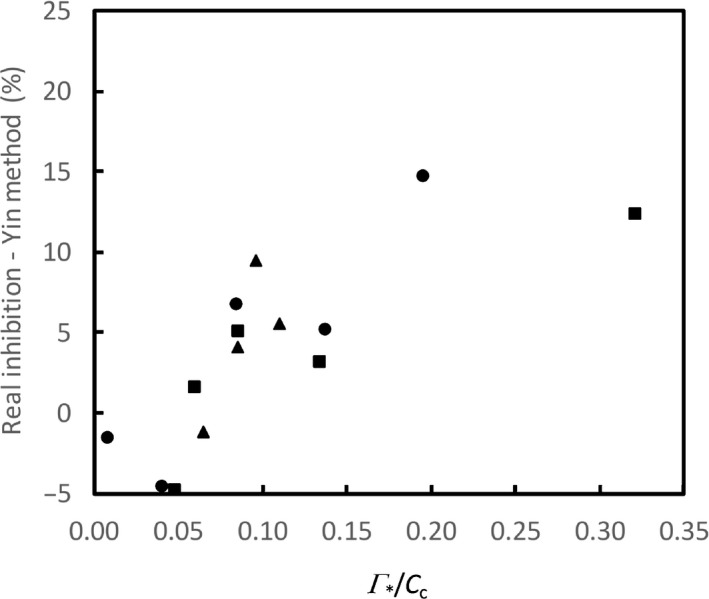
The relative real light inhibition of respiration identified by the Yin method plotted against ratios of CO_2_ compensation point to chloroplast CO_2_ concentration (Γ*/*C*_c_) across various O_2_ concentrations (circles), CO_2_ concentrations (squares), and temperatures (triangles) for sunflower leaves.

Compared with the relative apparent inhibition from the Yin method, the relative real inhibition was much lower (Fig. 6b). The results also suggested that after correcting for varying Γ*/*C*_c_, light inhibition of *R*_d_ only became lower but did not disappear: the real inhibition increased generally with relative amounts of photorespiration (Fig. 7).

## Discussion

### The ‘linear decrease’ of *R*_d_ with light cannot generate the Kok effect

The Kok effect was initially, and is still often, hypothesized to arise from the suppression of respiration by light (Fig. 1; Sharp *et al*., 1984; Heskel *et al*., 2013; Tcherkez *et al*., 2017a; Way *et al*., 2019). This hypothesis has received support from studies that have identified several mechanisms for the metabolic downregulation of respiratory reactions by light, as reviewed by Tcherkez *et al*. (2012, 2017b). Gas exchange measurements have shown that *R*_d_, relative to *R*_dk_, progressively decreased with increasing *I*_abs_, either in a continuously linear manner (Villar *et al*., 1994) or in a decelerating manner (Brooks & Farquhar, 1985; Villar *et al*., 1995; Atkin *et al*., 2000). Using Eqns (Eqn 1), (Eqn 2), (Eqn 3), we assessed the effect of all possible scenarios for the often‐said ‘progressive’ inhibition of respiration by light on the shape of *A*−*I*_abs_ curves (Fig. 3). Of the six scenarios considered, only the three scenarios for ‘decelerating decrease’ of *R*_d_ with irradiance (Fig. 3b–d) can generate the Kok effect, thereby excluding the other three scenarios that are often considered relevant to the Kok effect. In particular, the scenario of a ‘continuously linear decrease’ of *R*_d_ with light (Fig. 3a) did not result in a break in the linear *A*−*I*_abs_ relationship. This is in contrast to the statement of Tcherkez *et al*. (2017a) in their report for the 18^th^ New Phytologist Workshop that ‘the widely‐accepted (historical) origin of the Kok effect is the inhibition of respiratory metabolism by light (linear decrease of *R*_d_ with light)’. Given the consequences of the various scenarios on the Kok effect, and thus also on the estimation of Φ_CO2_, future studies should aim to reveal which of the three scenarios in Fig. 3(b)–(d) is most likely for the light inhibition of *R*_d_.

### Several mechanisms co‐contribute to the Kok effect

Our analyses suggest that not a single mechanism determines the Kok effect, but at least three mechanisms (i.e. decreasing Φ_2_ with irradiance, varying Γ*/*C*_c_, and light inhibition of *R*_d_) co‐contribute to it. Using a model, we quantitatively estimated the relative contribution of the CO_2_‐specific processes like refixation (reflected via Γ*/*C*_c_) vs the light‐dependent decrease in photochemical efficiency (Φ_2_) in explaining the Kok effect. Our result suggested that varying Γ*/*C*_c_ explained *c*. 25% of the Kok effect, while variable Φ_2_ cannot be ignored and explained *c*. 12% of the Kok effect, across various CO_2_, O_2_ and temperature conditions. The appreciable contribution of variable Φ_2_ is supported by decreases in the slope of phase 2, compared with Phase 1, of the *A*−*I*_abs_ plots under conditions where photorespiration is greatly suppressed, for example, for C_3_ species under low‐O_2_ conditions or for C_4_ species (Yin *et al*., 2011a).

However, there are still small decreases in the slope of phase 2 for C_3_ species under low‐O_2_ conditions or for C_4_ species when *A* was plotted against *I*_abs_Φ_2_ (Yin *et al*., 2011a). This effect in C_4_ species may reflect the low efficacy of the CO_2_‐concentrating mechanism (CCM) caused by a high leakiness at low irradiances (Kromdijk *et al*., 2010; Yin *et al*., 2011b). However, for a C_3_ species, Buckley *et al*. (2017) observed even more significant changes for developing leaves under 2% than under 21% O_2_ conditions, suggesting an involvement of other mechanisms. A fourth mechanism was shown here to potentially contribute to the Kok effect (Fig. 2d), but we were not able to verify it, as any variable *f*_aet_ and/or *ρ*_2_ are hard to identify at the light intensities showing the Kok effect. Our results, that *B*_1_ : *B*_2_ ratios (Table 2) and *R*_D1_ : *R*_D2_ ratios (Table 3) were very close to 1 at 2% O_2_ or 700 µmol mol^−1^ CO_2_ suggest that significant involvement of a fourth mechanism was highly unlikely. Thus, our remaining unexplained part (*c*. 63%) of the Kok effect is most likely a result of the light suppression of *R*_d_, in agreement with the statement of Buckley *et al*. (2017) on the dominant role of this third mechanism.

### A dual effect of photorespiration in contributing to the Kok effect

Our strong linear relationships between the *B*_1_ : *B*_2_ ratio and the Γ*/*C*_c_ ratio (Fig. 5a) confirmed previous results in the literature (Cornic & Jarvis, 1978; Ishii & Murata, 1972; Ishii & Schmid, 1981; Sharp *et al*., 1984; Farquhar & Busch, 2017; Way *et al*., 2019) showing that the Kok effect was strongly associated with the occurrence of photorespiration. Perhaps it is because of the significant contribution of varying Γ*/*C*_c_ that the Kok effect reported in the earlier days (Kok, 1949; Ishii & Schmid, 1981; Sharp *et al*., 1984) generally had sharper transition than the recent data (Farquhar & Busch, 2017; Tcherkez *et al*., 2017a; Way *et al*., 2019) because *C*_a_ has been increasing over years. However, the contribution of other factors as discussed earlier means that the Kok effect will never disappear in the future high‐CO_2_ atmosphere; instead, it will continue, but to a lesser extent.

Our modelling analysis suggests that strong associations between the *B*_1_ : *B*_2_ ratio and the Γ*/*C*_c_ ratio shown in Fig. 5(a) are the combined result of a dual effect of photorespiration in contributing to the Kok effect. The first‐type effect is what Farquhar & Busch (2017) discussed on the role of increasing Γ*/*C*_c_ with irradiance in explaining the Kok effect, as a result of regulation of *g*_s_ and *g*_m_. The second‐type effect is what we found here – the light inhibition of *R*_d_ identified after removing the first‐type effect was still positively correlated with Γ*/*C*_c_ (Fig. 7). Our results suggest that the second‐type effect, representing real biological inhibitions, probably contributed more to the Kok effect than the first‐type effect.

Farquhar & Busch (2017) demonstrated that the first‐type effect of photorespiration on the Kok effect can generate the apparent light inhibition of *R*_d_ for photorespiratory conditions. As stated in the introduction, this inhibition via regulation of *g*_s_ and *g*_m_ is the same as the importance that Loreto *et al*. (2001) emphasized for accounting for the refixation of respired CO_2_ when estimating *R*_d_. Loreto *et al*. (2001) stated that there would be no significant difference between *R*_d_ and *R*_dk_ if the refixation of respiratory CO_2_ during illumination is taken into account. Our results showing that, after correcting for varying Γ*/*C*_c_, light inhibition of *R*_d_ only became lower but did not disappear (Fig. 6b), do not agree with the conclusion of Loreto *et al*. (2001). The refixation is an important means to reduce the (photo)respiratory loss under photorespiratory conditions, but its net contribution to total photosynthesis should be negligible under nonphotorespiratory conditions (Yin *et al*., 2020). Berghuijs *et al*. (2019) showed that *R*_d_ estimated by the Kok method was closer to the estimate made by their model (that accounted for the refixation) under nonphotorespiratory than under photorespiratory conditions. The experiment of Loreto *et al*. (2001) was conducted with maize, a C_4_ species where Rubisco is expected to be surrounded by a high CO_2_ partial pressure as a result of the C_4_ CCM, and thus the refixation of CO_2_ released from respiration and photorespiration should have little contribution to the total assimilation. Using ^14^C‐labelling, Pärnik & Keerberg (1995, 2007a,b) showed that light inhibition of *R*_d_ occurs even when accounting for CO_2_ refixation. Gong *et al*. (2015) reported a high suppression of *R*_d_ by light in a C_4_ species. If refixation does occur appreciably in C_4_ species as Loreto *et al*. stated, it may reflect the refixation more by phospho*enol*pyruvate carboxylase than by Rubisco, which might contribute to leakiness.

### Apparent vs real light inhibition of *R*_d_

The suppression of *R*_d_ by light has been identified using the Kok method, in many experimental studies, including recent reports based on CO_2_‐exchange measurements (e.g. Buckley *et al*., 2017) or both CO_2_‐ and O_2_‐exchange measurements (e.g. Gauthier *et al*., 2018). Light is known to suppress the activity of enzymes that involve CO_2_‐releasing pathways contributing to *R*_d_ (Buckley & Adams, 2011; Tcherkez *et al*., 2012, 2017a,b). Using the model analysis, Farquhar & Busch (2017) demonstrated that at least part of the light inhibition of *R*_d_ can be generated without assuming this inhibition beforehand. Here we used the modelling approach to analyse combined CO_2_‐exchange and Chl fluorescence data. With such combined experimental and modelling analyses, we demonstrated quantitatively that the original Kok method that attributes the Kok effect entirely to the light inhibition of *R*_d_ overestimated the real inhibition (Fig. 6), as a result of ignoring the contribution of varying Φ_2_ and Γ*/*C*_c_ to the Kok effect. The effect of varying Φ_2_ on the Kok method in overestimating the inhibition has been corrected simply by the Yin method, while the correction for varying Γ*/*C*_c_ is more complicated. We previously stressed that both Kok and Yin methods to estimate *R*_d_ actually apply to nonphotorespiratory conditions (Yin *et al*., 2011a). Our analysis with Eqn 5 suggests an approach to estimate the real light suppression of *R*_d_ for photorespiratory conditions, although we are unable to clarify which one of the three scenarios of suppression in Fig. 3(b–d)) is most likely. Most importantly, our analysis using Eqn 5 revealed that the real suppression still increased with relative amounts of photorespiration (Fig. 7). While this new empirical trend receives the support from a theoretical analysis of Buckley & Adams (2011) that photorespiratory NADH may be involved in the suppression, there are probably other underlying biochemical mechanisms that merit further investigation.

## Author contributions

XY conceived the study, XY and PELvdP designed the experiment, YN and PELvdP implemented the experiment and conducted the measurements, XY and YN analysed the data, and XY wrote the draft and finalised it with significant input from PCS.

## Supporting information


**Fig. S1** Photosystem II photochemical efficiency (Φ_2_) as a function of absorbed irradiance (*I*_abs_) across various O_2_ and CO_2_ concentrations and various temperatures.
**Fig. S2** Comparison of net photosynthesis rate *A* and the average Γ*/*C*_c_ ratio modelled using three mesophyll conductance *g*_m_ modes as described in the text.
**Table S1** List of all model symbols.
**Table S2** Model parameter values estimated using three mesophyll conductance *g*_m_ modes.
Please note: Wiley Blackwell are not responsible for the content or functionality of any Supporting Information supplied by the authors. Any queries (other than missing material) should be directed to the *New Phytologist* Central Office.
