# Supplementary material for: The Kok effect revisited
Source: New Phytol. 2020 Jun 3;227(6):1764–75. doi: 10.1111/nph.16638 (PMC7497127; doi:10.1111/nph.16638)
Supplement: Supplementary file 1 — Fig. S1 Photosystem II photochemical efficiency (Φ2) as a function of absorbed irradiance (I abs) across various O2 and CO2 concentrations and various temperatures. Fig. S2 Comparison of net photosynthesis rate A and the average Γ*/C c ratio modelled using three mesophyll conductance g m modes as described in the text. Table S1 List of all model symbols. Table S2 Model parameter values estimated using three mesophyll conductance g m modes. Please note: Wiley Blackwell are not responsible for the content or functionality of any Supporting Information supplied by the authors. Any queries (other than missing material) should be directed to the New Phytologist Central Office. [file NPH-227-1764-s001.pdf]

New Phytologist

***New Phytologist* Supporting Information**

Article title: The Kok effect revisited
Authors: Xinyou Yin, Yuxi Niu, Peter E.L. van der Putten, Paul C. Struik
Article acceptance date: 24 April 2020

The following Supporting Information is available for this article:

Fig. S1 Photosystem II photochemical efficiency (Φ_2) as a function of absorbed irradiance (I_{abs}) across various O_2, CO_2 and temperature levels

Fig S2 Comparison of net photosynthesis rate A and the average Γ_*/C_c ratio modelled using three g_m-modes as described in the text

Table S1 List of all model symbols

Table S2 Values of model parameters estimated using the three g_m-modes

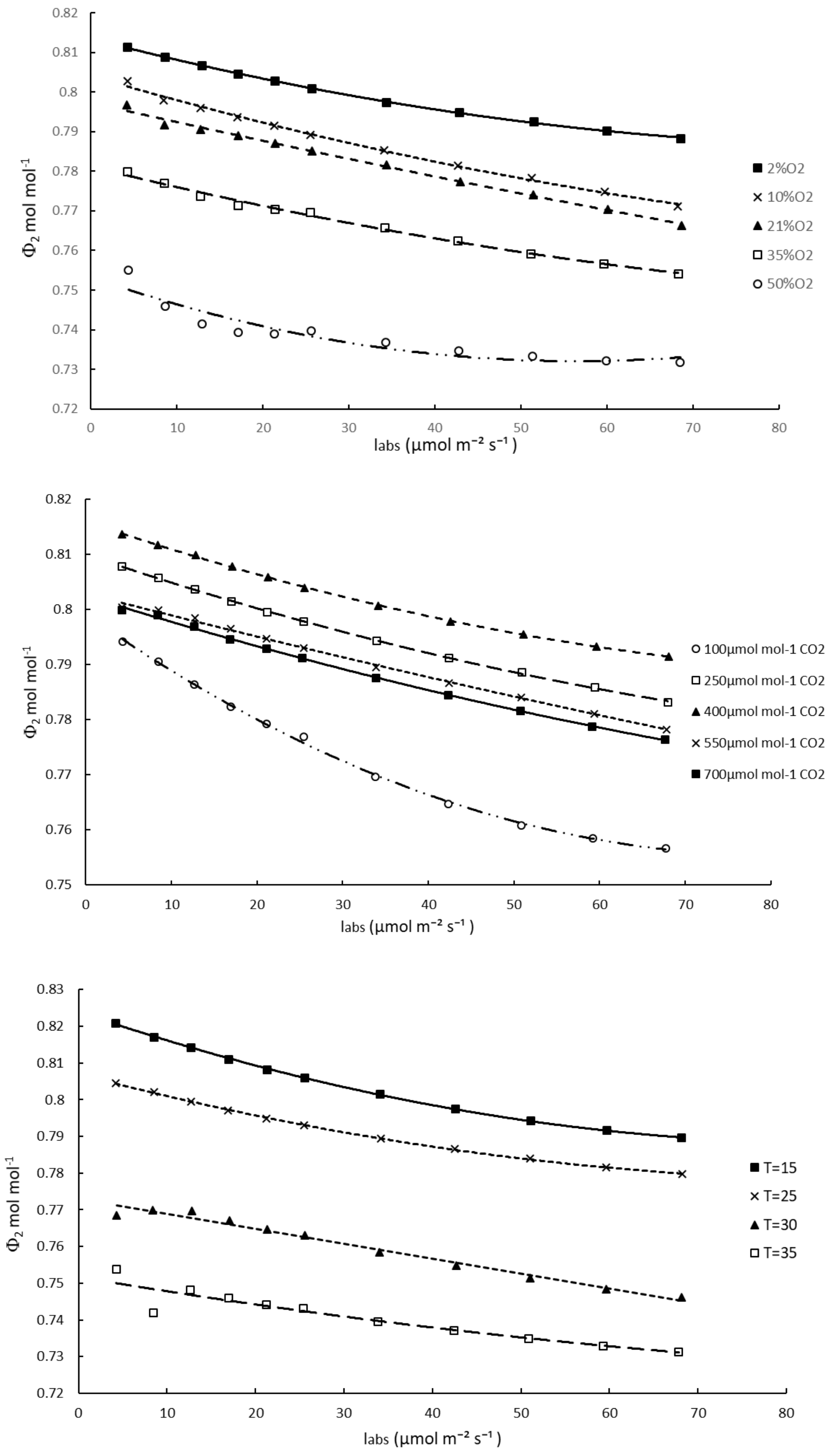

Fig. S1 PSII photochemical efficiency (Φ_2) as a function of absorbed irradiance (I_{abs}) across various O_2, CO_2 and temperature levels. Points represent the mean of measurements on four replicated sunflower leaves.

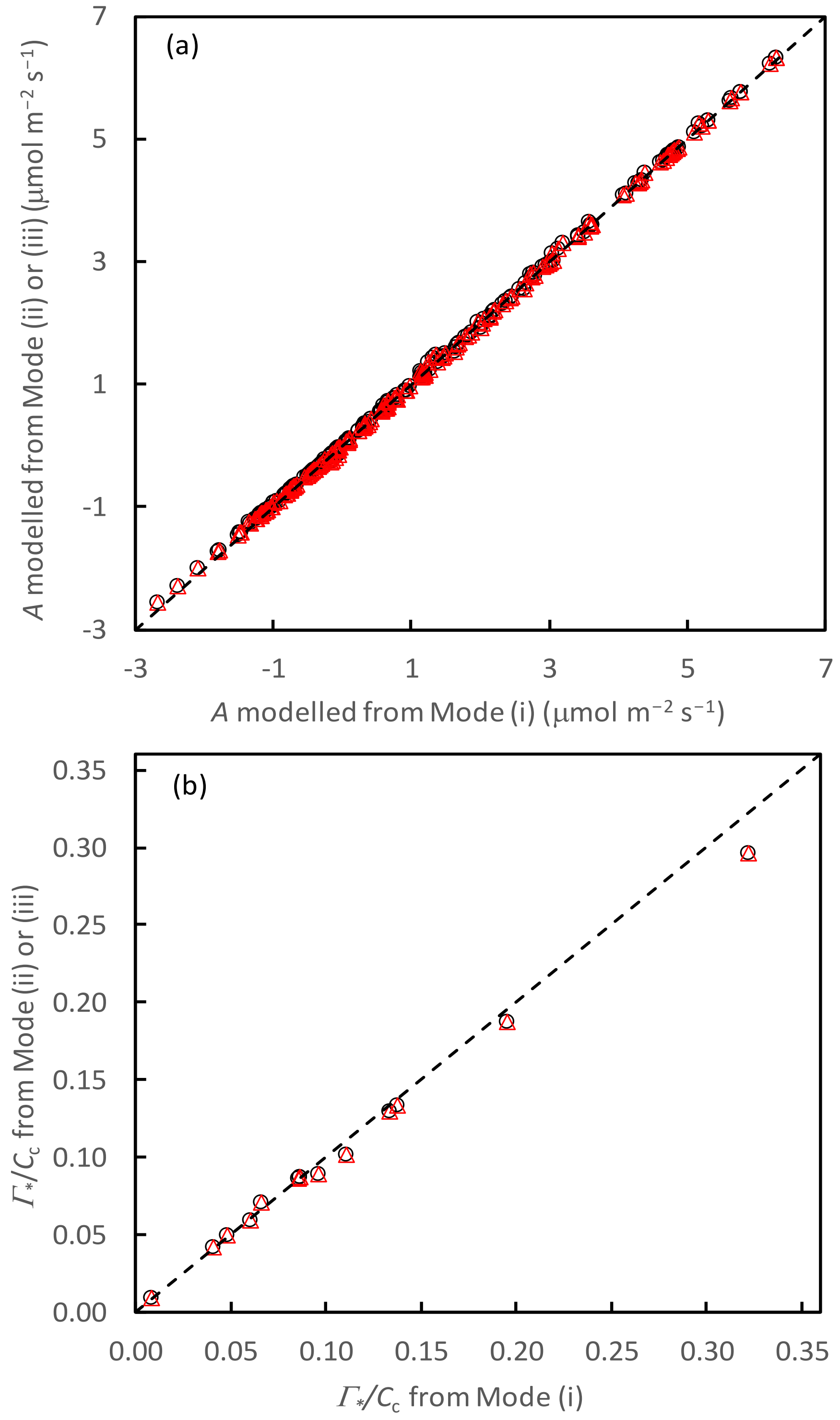

Figure S2 Comparison of sunflower net photosynthesis rate A (panel a) and the average Γ_*/C_c ratio (panel b) modelled using the g_m-Mode (ii) (black circles) or Mode (iii) (red triangles) versus those modelled using the g_m-Mode (i). The three g_m-Modes are as described in the text. The dashed diagonal represents the 1:1 line, at which $y = x$. See Table S1 for symbol definition.

Table S1 List of all model symbols

Symbol	Definition	Unit
A	Net leaf photosynthesis	μmol m^{-2} s^{-1}
b_1	Slope of Phase 1 (below the transition point) in the A versus I_{abs} plot	mol mol^{-1}
b_2	Slope of Phase 2 (above the transition point) in the A versus I_{abs} plot	mol mol^{-1}
B_1	Slope of Phase 1 (below the transition point) in the A versus $I_{abs}\Phi_2$ plot	mol mol^{-1}
B_2	Slope of Phase 2 (above the transition point) in the A versus $I_{abs}\Phi_2$ plot	mol mol^{-1}
C_a	Ambient CO_2 level	μmol mol^{-1}
C_c	Chloroplast CO_2 level at the carboxylating sites of Rubisco	μbar
C_i	Intercellular CO_2 level	μbar
f_{aet}	Fraction for alternative electron transport, defined by eqn (2)	-
f_{cyc}	Fraction of total PSI electron flux that follows cyclic electron transport	-
f_{pseudo}	Fraction of total PSI electron flux that follows pseudocyclic electron transport	-
g_m	Mesophyll conductance for CO_2 transfer	mol m^{-2} s^{-1} bar^{-1}
g_{m30}	Mesophyll conductance for CO_2 transfer at 30°C	mol m^{-2} s^{-1} bar^{-1}
g_s	Stomatal conductance for CO_2 transfer	mol m^{-2} s^{-1}
I_{abs}	Irradiance absorbed by leaf photosynthetic pigments	μmol m^{-2} s^{-1}
$I_{abs,t}$	The level of I_{abs}, at which the transition from Phase 1 to Phase 2 takes place	μmol m^{-2} s^{-1}
I_{inc}	Incident Irradiance	μmol m^{-2} s^{-1}
J	Linear electron transport rate (= $s\Phi_2 I_{inc}$; see the text)	μmol m^{-2} s^{-1}
m	A factor that lumps several parameters in a multiple mesophyll-resistance model	-
O	Oxygen level	mbar
R_d	Day respiration (i.e. CO_2 release from all processes other than photorespiration)	μmol m^{-2} s^{-1}
r_{d1}	Intercept of Phase 1 (below the transition point) in the A versus I_{abs} plot	μmol m^{-2} s^{-1}
r_{d2}	Intercept of Phase 2 (above the transition point) in the A versus I_{abs} plot	μmol m^{-2} s^{-1}
R_{D1}	Intercept of Phase 1 (below the transition point) in the A versus $I_{abs}\Phi_2$ plot	μmol m^{-2} s^{-1}
R_{D2}	Intercept of Phase 2 (above the transition point) in the A versus $I_{abs}\Phi_2$ plot	μmol m^{-2} s^{-1}
R_{dk}	Respiration in darkness	μmol m^{-2} s^{-1}
s	Calibration factor that converts chlorophyll fluorescence-based Φ_2 into J	-
$S_{c/o(25)}$	Relative CO_2/O_2 Specificity of Rubisco (at 25°C)	mbar $μbar^{-1}$
δ	Coefficient in g_m-Mode (ii) or (iii) (see the text), implicitly defining variable g_m	-
Φ_1	Photochemical efficiency of PSI electron transport	mol mol^{-1}
Φ_2	Photochemical efficiency of PSII electron transport	mol mol^{-1}
Φ_{CO2}	Quantum efficiency of CO_2 assimilation	mol mol^{-1}
Γ_*	The C_c level at which carboxylation equals photorespiratory CO_2 release	μbar
ρ_2	Fraction of I_{abs} that is partitioned to PSII	-
Ω	Gap between 30°C and the temperature at which g_m falls to e^{-1} of its maximum	°C

Table S2 Values (standard error of the estimate in brackets) of model parameters estimated for sunflower leaves using the three g_m-modes as described in the main text

Parameter [a]	Mode (i)	Mode (ii)	Mode (iii) [b]
s	0.4259(0.0019)	0.4312(0.0022)	0.4312(0.0022)
$S_{c/o25}$ (mbar μbar^{-1})	3.320(0.060)	3.211(0.045)	3.474(0.086)
g_{m30} (mol m^{-2} s^{-1} bar^{-1}) [c]	0.145(0.030)	n.a.	n.a.
Ω (°C)	30.9(13.88)	n.a.	n.a.
δ	n.a.	5.67(1.09)	3.67(0.76)
R^2	0.996	0.996	0.996

[a] See Table S1 for parameter definitions;

[b] assuming that the *m* factor = 0.3 (see the text);

[c] for comparison with the g_m estimate: stomatal conductance for CO_2 diffusion (g_s) varied with measurement conditions but its overall average was 0.175 mol m^{-2} s^{-1};

n.a., not applicable.
